# CoQ_10_ Deficient Endothelial Cell Culture Model for the Investigation of CoQ_10_ Blood–Brain Barrier Transport

**DOI:** 10.3390/jcm9103236

**Published:** 2020-10-10

**Authors:** Luke Wainwright, Iain P. Hargreaves, Ana R. Georgian, Charles Turner, R. Neil Dalton, N. Joan Abbott, Simon J. R. Heales, Jane E. Preston

**Affiliations:** 1 UCL Queen Square Institute of Neurology, University College London, London WC1N 3BG, UK; luke.wainwright.14@ucl.ac.uk; 2 Neurometabolic Unit, National Hospital for Neurology and Neurosurgery, University College London Hospitals NHS Foundation Trust, London WC1N 3BG, UK; i.p.hargreaves@ljmu.ac.uk; 3 Department of Pharmacy and Biomolecular Science, Liverpool John Moores University, Liverpool L3 5UA, UK; 4 School of Cancer and Pharmaceutical Sciences, King’s College London, London SE1 9NH, UK; ana.georgian@kcl.ac.uk (A.R.G.); joan.abbott@kcl.ac.uk (N.J.A.); 5 Evelina London Children’s Hospital, Guy’s and St. Thomas’ NHS Foundation Trust, London SE1 7EH, UK; charles.turner@gstt.nhs.uk (C.T.); neil.dalton@gstt.nhs.uk (R.N.D.); 6 UCL Great Ormond Street Institute of Child Health, University College London, London WC1E 6BT, UK; s.heales@ucl.ac.uk

**Keywords:** coenzyme Q_10_, coenzyme Q_10_ deficiency, blood–brain barrier, bEND.3, PBEC, mitochondrial dysfunction, lipoprotein, LDLR, RAGE, SR-B1, LC-MS/MS

## Abstract

Primary coenzyme Q_10_ (CoQ_10_) deficiency is unique among mitochondrial respiratory chain disorders in that it is potentially treatable if high-dose CoQ_10_ supplements are given in the early stages of the disease. While supplements improve peripheral abnormalities, neurological symptoms are only partially or temporarily ameliorated. The reasons for this refractory response to CoQ_10_ supplementation are unclear, however, a contributory factor may be the poor transfer of CoQ_10_ across the blood–brain barrier (BBB). The aim of this study was to investigate mechanisms of CoQ_10_ transport across the BBB, using normal and pathophysiological (CoQ_10_ deficient) cell culture models. The study identifies lipoprotein-associated CoQ_10_ transcytosis in both directions across the in vitro BBB. Uptake via SR-B1 (Scavenger Receptor) and RAGE (Receptor for Advanced Glycation Endproducts), is matched by efflux via LDLR (Low Density Lipoprotein Receptor) transporters, resulting in no “net” transport across the BBB. In the CoQ_10_ deficient model, BBB tight junctions were disrupted and CoQ_10_ “net” transport to the brain side increased. The addition of anti-oxidants did not improve CoQ_10_ uptake to the brain side. This study is the first to generate in vitro BBB endothelial cell models of CoQ_10_ deficiency, and the first to identify lipoprotein-associated uptake and efflux mechanisms regulating CoQ_10_ distribution across the BBB. The results imply that the uptake of exogenous CoQ_10_ into the brain might be improved by the administration of LDLR inhibitors, or by interventions to stimulate luminal activity of SR-B1 transporters.

## 1. Introduction

Coenzyme Q_10_ (CoQ_10_) plays an important role in oxidative phosphorylation where it acts as an electron carrier in the mitochondrial respiratory chain (MRC). Its major role is in accepting electrons derived from complex I and II (NADH ubiquinone reductase; succinate ubiquinone reductase) and transporting them to complex III (ubiquinol cytochrome c reductase) [1]. In addition to this, CoQ_10_ also serves as an antioxidant and an essential component in the functional superassembly of the so-called “respirasome” [2], which improves efficiency and prevents electron leakage and production of reactive oxygen species (ROS).

CoQ_10_ deficiencies are defined by decreased cellular CoQ_10_ content, and pathogenesis involves both reduced ATP production and increased ROS production [3]. Primary CoQ_10_ deficiencies stem from mutations in genes required for CoQ_10_ biosynthesis (nine genes have been identified [3]) while secondary deficiencies are associated with diseases that do not result from a genetic defect in the CoQ_10_ biosynthetic pathway and include disorders such as primary MRC deficiencies and organic acidemias [4]. A failure in CoQ_10_ biosynthesis could therefore contribute to disease pathophysiology by causing a failure in energy metabolism and/or increased oxidative stress.

The clinical presentation of CoQ_10_ deficiency is heterogeneous, however, there are five distinct clinical phenotypes: encephalomyopathy; severe infantile multisystemic disease; nephropathy; cerebellar ataxia and isolated myopathy [5]. Since the first description of human CoQ_10_ deficiency in 1989, over 150 cases have been reported, with cerebellar ataxia being the most common clinical presentation of this disorder [5]. Many patients respond well to oral supplementation of high dose CoQ_10_ which can stop the progression of the encephalopathy [6,7]. However, in other patients with predominantly central nervous system (CNS) manifestations including ataxia, seizures or dystonia characterised by recessive ADCK3 mutations, which encodes for a protein kinase that is involved in CoQ_10_ biosynthesis and its regulation [8], there is often no or limited clinical benefit of CoQ_10_ supplementation [9]. Indeed, only 49% of patients with the cerebellar ataxic phenotype have been reported to demonstrate improvement/stabilisation in their ataxic symptoms following CoQ_10_ supplementation [5].

The reasons for the refractory nature of these neurological symptoms to CoQ_10_ supplementation remain unknown. However, a major contributory factor may be the poor transfer of CoQ_10_ across the blood–brain barrier (BBB) into CNS, resulting in insufficient CoQ_10_ availability for the deficient neurons. Relatively little is known about how plasma CoQ_10_ interacts with the BBB or whether CoQ_10_ deficiency of the BBB itself may affect transport into the central nervous system. Plasma CoQ_10_ is in the form of reduced CoQ_10_H_2_, or ubiquinol, and carried by lipoproteins including HDL, LDL, vLDL [10], following absorption in the small intestine and processing by the liver [11]. Lipoproteins serve to solubilise lipophilic compounds such as CoQ_10_, and transport them through the aqueous circulatory system. Specific apoproteins present on the surface of the macromolecule facilitate their targeting to appropriate tissues by receptor-mediated endocytic processes.

At the BBB, there are multiple membrane transporters for uptake and efflux that interact with lipoproteins [12], but these generally act together to limit systemic lipoprotein transfer into the brain. An exception is HDL which traverses the BBB by caveolin-mediated transcytosis after interaction with the SR-B1 scavenger receptor on the apical (blood side) of the BBB [13]. Under normal circumstances, systemic lipoprotein and cholesterol are not required by the brain because there is sufficient *de novo* synthesis, mainly by astrocytes [14].

In this study, we assessed the permeability of an in vitro BBB model to CoQ_10_ and the effect of induced CoQ_10_ deficiency on transport. Using pharmacological inhibitors of BBB lipoprotein transporters, we also investigated their effect on CoQ_10_ transport, specifically, BLT-1 inhibitor of SR-B1 (Scavenger Receptor) mediated HDL uptake [15], the receptor-associated protein (RAP) inhibitor of the Low-Density Lipoprotein Receptor (LDLR) superfamily [16], including LRP-1, vLDLR, apoER2, and LDLR; and FPS-ZM1 inhibitor of the receptor for advanced glycation end products (RAGE) which opposes LRP-1 as part of apolipoprotein E-amyloid beta homeostasis [17]. In addition, the efflux transporter P-glycoprotein (ABCB1) was studied since it is reported to reduce CoQ_10_ transport across the Caco-2 intestinal epithelial-barrier model [18].

## 2. Experimental Section

### 2.1. Materials

Unless otherwise stated all materials were supplied by Sigma-Aldrich Company Ltd., UK.

### 2.2. CoQ_10_ Analysis by Liquid Chromatography-Tandem Mass Spectrometry

A novel CoQ_10_ liquid chromatography-tandem mass spectrometry (LC-MS/MS) method was established. The method is a modified version of that described by Itkonen et al. [19], in combination with a variation of the sample preparation outlined by Duncan et al. [20]. The lower limit of quantitation for this method is 0.25 nmol/L, with a limit of detection at 0.125 nmol/L, and linearity up to 500 nmol/L. The run-time (inject-to-inject) is 7 min per sample.

Samples were prepared by the addition of stable isotope-labelled internal standard (CoQ_10_–[^2^H_9_]; IsoSciences LLC, Ambler, PA, USA) to each sample (200 μL), with a subsequent freeze–thaw process (×3) to perturb cellular membranes. Extraction buffer was then added (800 µL/sample; 5:2 (*v/v*) hexane/ethanol) and the samples vigorously mixed on a vortex for 1 min, centrifuged at 18,625× *g* for 3 min, and the top layer of hexane collected. The hexane extract was evaporated to dryness using a centrifugal evaporator. Prior to analysis, calibrators and samples were re-constituted in LC-MS/MS “running solvent A” (50 µL; 41:9 (*v/v*) methanol/1-propanol with 500 µmol/L ammonium acetate), vigorously mixed, and transferred into a suitable vial. CoQ_10_ calibration curves (0, 0.25, 0.5, 1.0, 2.5, 5.0, 25, 50, 500 nmol/L CoQ_10_ in ethanol) were established through serial dilutions of a 1 mM stock solution, as confirmed by the spectrophotometric method first described by Crane et al. [21].

Chromatography was performed on an Agilent Technologies 1200 Series LC system (Agilent Technologies, USA) using an ACE^®^ UltraCore™ 2.5 µm SuperC18™ 30 × 2.1 mm reversed-phase column (Advanced Chromatography Technologies Ltd., Aberdeen, UK) kept at 25 °C with a gradient of running solvent A (41:9 (*v/v*) methanol/1-propanol with 500 µmol/L ammonium acetate) and running solvent B (1:1 (*v/v*) methanol/1-propanol with 500 µmol/L ammonium acetate). The gradient elution profile was maintained at 100% A (0–0.2 min), ramped to 100% B (0.21–1 min), maintained at 100% B (1–3.5 min), and ramped back to 100% A (3.51–3.6 min). Total run time was 6.5 min with a flow rate of 220 µL/min and injection volume of 10 µL.

Mass spectrometry was performed on an AB Sciex™ QTRAP^®^ 6500 (ESI)-MS/MS (AB Sciex™, Macclesfield, UK), operated in positive ion mode with the ion source spray voltage at 5500 V, declustering potential at 50 V, temperature at 115 °C, and collision energy at 27 V. The curtain gas was 48 L/min, gas 1 (nebuliser gas) 55 L/min, gas 2 (heater gas) 21 L/min, and collision gas on “medium” setting. The mass spectrometer was programmed to monitor the transitions of *m/z* 880.7–197.1 (dwell time 200 ms) corresponding to the ammonium adduct of CoQ_10_, and *m/z* 889.7–206.1 (dwell time 200 ms) corresponding to the ammonium adduct of CoQ_10_–[^2^H_9_].

Final CoQ_10_ concentrations (nmol/L) were calculated as a ratio of CoQ_10_/CoQ_10_–[^2^H_9_] peak areas, and quantified against the corresponding calibration curve, with appropriate correction for dilution. For intracellular determination of CoQ_10_ the concentration was divided by total protein (mg/mL) and expressed as pmol/mg (nmol/g) of protein.

### 2.3. Blood–Brain Barrier Cell Culture

Two BBB cell culture models were used in this study. The bEND.3 cell line is a widely characterised, consistent, and easy to use in vitro BBB model, which was used as the main tool to interrogate lipoprotein-CoQ10 transport. Key findings using bEND.3 cells were then replicated using primary porcine brain endothelial cells for validation.

Primary porcine brain endothelial cells (PBEC) were isolated and cultured as previously published [22]. Briefly, cells were seeded at 10 × 10^4^ cells/cm^2^ on collagen and fibronectin-coated polycarbonate Transwell filters (Corning 0.4 µm pore size) in 12-well plates, and grown in low glucose DMEM (Sigma D5546) supplemented with bovine plasma-derived serum (10% *v/v*; BPDS, First Link UK), glutamine (2 mM), heparin (125 µg/mL), penicillin (100 U/mL) and streptomycin (100 µg/mL), at 37 °C in a 5% CO_2_ incubator. PBECs were grown in non-contact co-culture above primary rat astrocytes until confluent, then supplemented with hydrocortisone (550 nM), 8-4-chlorophenylthio-cAMP (250 µM) and RO-20-1724 (17.5 µM) in serum-free medium for a further 3 days. Before assays of CoQ_10_ transport, PBECs were separated from astrocytes by moving transwell filters to fresh culture plates. PBEC monolayer tightness was assessed by transendothelial electrical resistance (TEER, STX100C Electrode) and FITC-dextran 40 (FITC-40) paracellular permeability (P*app*), as previously described by Patabendige et al. [23]. The TEER averaged 946 ± 94 Ω · cm^2^ (after subtraction of blank 160 Ω · cm^2^) and FITC-40 P*app* averaged 1.3 ± 0.1 × 10^−6^ cm · sec^−1^ (*n* = 12). High TEER and low paracellular permeability demonstrate a tight BBB monolayer.

The mouse BBB cell line, bEnd.3 (ATCC CRL-2299) was used for pharmacological screening of potential transport system inhibitors. Cells, between passage 24–28, were seeded at 2.5 × 10^4^ cells/cm^2^ onto collagen-coated Transwell filters (Corning 0.4 µm pore size) in 12 well plates, and grown in DMEM (ATCC, 30-2002) with foetal bovine serum (10% *v/v*), penicillin (100 U/mL) and streptomycin (100 µg/mL) until confluent. The TEER and FITC-40 P*app* averaged 40.8 ± 3.2 Ω · cm^2^ (after subtraction of blank 160 Ω · cm^2^) and 3.3 ± 0.4 × 10^−6^ cm.sec^−1^ (*n* = 12) respectively. The tight-junction integrity of bEnd.3 monolayers is lower when compared to PBEC, but suitable for assessing permeability of macro-molecules across a monolayer (e.g., lipoproteins) while less suitable for small molecules (~400 g/mol) due to paracellular leak.

### 2.4. Coenzyme Q_10_ Transport Assays

Cell medium was replaced by an assay buffer of HBSS (without phenol red), bovine serum albumin (BSA; 0.5% *w/v*), HEPES (25 mM) titrated to *p*H 7.4 and FITC-40 (1 mg/mL). Unless stated, CoQ_10_ was pre-treated by incubation in serum at a concentration of 20 µM for 45 min at 37 °C, and added to assay buffer on either the Apical (blood facing) or Basal (brain facing) side of the cells on Transwell filters. The final concentration of CoQ_10_ used in the assays was 10 µM in serum (50% *v/v*). Cells were then incubated for 60 min on an orbital shaker (100 rpm) at 37 °C. Samples of the Apical (A) and Basal (B) media were then taken for analysis to calculate A to B (blood-to-brain) or B to A (brain-to-blood) P*app* in cm/s, as previously described by Patabendige et al. [23]. CoQ_10_ concentrations were determined using the LC-MS/MS method described above (Section 2.2). FITC-40 was measured fluorometrically (excitation 485/20 nm, emission 528/20 nm, sensitivity 50) on a Synergy™ HT plate reader with KC4™ data analysis software (BioTek Instruments Ltd., Cheadle, UK).

Inhibitors of transport were added to both Apical and Basal sides of the cells for 2 h prior to assay. Antioxidants were pre-incubated with CoQ_10_ in serum for 45 min prior to assay, and were present in the assay buffer. Compounds used were; SR-B1 inhibitor, blocker of lipid transport-1 (BLT-1; 10 µmol/L) [15] LDLR superfamily inhibitor, RAP (0.5 µmol/L) [24]; RAGE inhibitor, FPS-ZM1 (1 µmol/L) [25]; P-glycoprotein inhibitor, verapamil 0.1 mmol/L [26]; α-tocopherol (vitamin E; 50 µmol/L) [27]; and Trolox (50 µmol/L).

### 2.5. Cellular CoQ_10_ Depletion and Mitochondrial Respiratory Chain Enzyme Activity

As previously described, *para*-aminobenzoic acid (*p*ABA) was used as a pharmacological reagent to induce CoQ_10_ deficiency [3,28,29]. The mechanism of action is via competitive inhibition of polyprenyl-4-hydroxybenzoate transferase (Coq2p), a key enzyme in the later stages of the CoQ_10_ biosynthetic pathway. Following the method of Duberley et al. [29], 1 mmol/L *p*ABA was added to culture medium for 5 days prior to assay.

Activities of the mitochondrial respiratory chain enzymes; complex I, complex II-III and complex IV together with the mitochondrial marker enzyme, and citrate synthase (EC 2.3.3.1) were determined spectrophotometrically on a Uvikon XL spectrophotometer with LabPower software (Version 2.06S, Northstar Scientific Ltd., Potton, UK) according to the method previously described by Hargreaves et al. [30]. Results were expressed as a ratio to citrate synthase activity, a validated biomarker of mitochondrial content, and were normalised against mg protein. Protein quantification was determined according to the Lowry method [31] using BSA as a standard.

### 2.6. Cell Viability Assay

Cell viability was determined using the MTT assay described by Mosmann [32]. Cells were passaged onto 96-well plates, grown to confluence and washed with HBSS prior to the addition of 3-[4,5-dimethylthiazol-2-yl]-2,5-diphenyl tetrazolium bromide (MTT; 1 mg/mL) in DMEM (without phenol red). Cells were then incubated for 4 h at 37 °C, 5% CO_2_, after which the medium was removed, and the remaining formazan crystals dissolved in propan-2-ol (100 μL/well). The resulting purple solution was spectrophotometrically measured at 540 nm using a Multiskan Ascent plate reader with Ascent software (MTX LabSystems, USA).

### 2.7. CoQ_10_ Partition in Serum Lipoprotein Fractions

Bovine plasma-derived serum (BPDS) was either untreated or supplemented with 10 µM CoQ_10_ for 45 min at 37 °C. Serum lipoproteins were then fractionated according to the method of Ononogbu et al. [33] and the CoQ_10_ content measured in each fraction by LC-MS/MS. This lipoprotein fraction method is comprised of two parallel precipitation-centrifugation extractions, yielding a separation of the major classes of lipoprotein as supernatants containing “LDL + HDL” and “HDL”. The concentration of CoQ_10_ in the “VLDL” fraction was calculated by subtraction.

### 2.8. Confocal Microscopy

Cells for confocal microscopy were fixed in paraformaldehyde (4% *w/v* in PBS) for 45 min, washed with HBSS and stored in glycerol (70% *v/v* in PBS) until use. Cells were permeabilised with Triton-x 100 (0.1% *v/v* in PBS), incubated with anti-Claudin 5 Monoclonal Antibody (4C3C2) Alexa Fluor 488 (1 in 80 dilution in DAKO, overnight, 4 °C, Thermofisher) to visualise tight junctions. Samples were then mounted in Vectashield containing DAPI for nuclei staining. The Nikon A1 inverted confocal microscope was used with spectral detector and Eclipse Ti-E microscope at ×40 magnification to generate digital images, analysed using Fiji (ImageJ).

### 2.9. Statistical Analysis

All results are expressed as mean ± standard error of the mean (SEM). Individual comparisons of means were made using the two-sample Student’s t-test and were carried out using Microsoft^®^ Excel with AnalystSoft^®^ StatPlus software (Version 5.4, Analyst Soft, Walnut, CA, USA). To reduce the incidence of type 1 error that is associated with performing multiple two-sample t-tests, one-way ANOVA was used for comparison of groups > 2, with Bonferroni post-hoc analysis. In all cases, *p* < 0.05 was considered significant.

## 3. Results

### 3.1. LC-MS/MS CoQ_10_ Method Validation

The lower limit of detection (LLOD) for the LC-MS/MS method was 0.125 nmol/L and defined as a signal-to-noise ratio of 3 (*n* = 6). Linearity and lower limit of quantitation (LLOQ) were determined across a 10-point serial dilution (0–500 nmol/L) performed on six separate days with six separate preparations, and defined as the lowest concentration and range, respectively, that could be measured with an inaccuracy (percentage relative error) and imprecision (CV%) < 20% (*n* = 6) [34,35]. For this method, the LLOQ was found to be 0.25 nmol/L with linearity up to 500 nmol/L. This performance surpasses the current HPLC-UV [20] technique which is commonly used for clinical diagnosis (Table 1). However, we did not compare this LC-MS/MS method with HPLC-electrochemical detection which is an analytical technique that can also be used for the clinical assessment of CoQ_10_ and has the ability to determine both the CoQ_10_ and ubiquinol species in tissues [36].

The precision of the LC-MS/MS method was assessed by evaluating the intra- and inter-assay coefficient of variation (CV), with acceptable CV values being defined as < 15% [34,35,37]. The intra-assay precision was determined across replicates of three parallel samples of internal QC (IQC) material (*n* = 8; baseline, low spike, high spike). Inter-batch precision was calculated as the CV of average values for parallel samples of QC material over seven separate days (*n* = 2; baseline, low spike, “plasma” QC). The results (Table 2) indicate that the LC-MS/MS method has good reproducibility across the range.

Accuracy was investigated by examining the average recovery of known quantities of CoQ_10_ in replicates of spiked samples (*n* = 8; low spike (+10 nmol/L), high spike (+100 nmol/L)). A negligible inaccuracy (3%) was observed for the high spike at 100 nmol/L. The relatively low, but consistent, recovery (84%) for the low spiked CoQ_10_ sample at 10 nmol/L could be due to adsorption losses during sample preparation, but overall the method exhibits an acceptable degree of accuracy across the range (Table 2).

Carry-over between successive samples was assessed by analysing a blank sample immediately after the highest calibrator standard (ULOQ; 500 nmol/L) (*n* = 7). No quantifiable carryover was observed for the LC-MS/MS method.

These results suggest that LC-MS/MS could be a viable alternative to current clinical techniques, namely HPLC-UV, and offers improved performance which could prove advantageous for the timely diagnosis of CoQ_10_ deficiencies in humans.

### 3.2. Effect of Serum Pre-Incubation on CoQ_10_ Transport across In Vitro BBB

To date, the highest achievable CoQ_10_ plasma concentration observed after oral-supplementation in vivo is 10.7 μmol/L [38,39], and treatment with 10 μmol/L CoQ_10_ restores MRC function in CoQ_10_ deficient human neuroblastoma cells [40]. Therefore, 10 μmol/L CoQ_10_ was selected as the clinically relevant concentration for use in this study.

**Figure 1:**
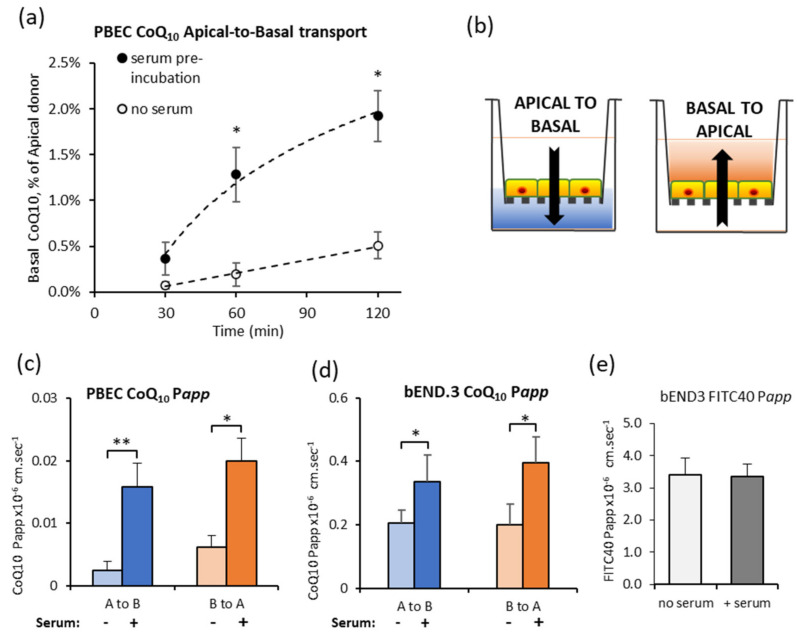
Effect of pre-incubating CoQ_10_ in serum, on transport across the blood–brain barrier (BBB) in vitro models. (**a**) CoQ_10_ transport across porcine brain endothelial cells (PBEC) monolayer is increased by pre-incubation of CoQ_10_ in serum. CoQ_10_ was either pre-incubated for 45 min in serum, or added directly to apical assay buffer (no serum), and appearance in the basal buffer measured after 30, 60 or 120 min; *n* = 4–7. (**b**) Schematic of transport assays using BBB cells grown on Transwell filters. CoQ_10_ and FITC-40 can be added to the apical compartment to measure transport from blood-to-brain, (apical to basal), or to the basal compartment to measure brain-to-blood transport (basal to apical). CoQ_10_ P*app* across PBEC (**c**) and bEnd.3 (**d**) monolayers after 60 min. Transport in both directions, blood-to-brain side (A to B) and brain-to-blood side (B to A) was increased after pre-incubation of CoQ_10_ in serum; *n* = 4–10. (**e**) FITC-40 P*app* across b.End3 monolayer over 60 min. Pre-incubation of FITC-40 in serum did not change transport in either direction; *n* = 6–12. Values are mean ± SEM; * *p* < 0.05, ** *p* < 0.01.

The time course for CoQ_10_ transport across the primary PBEC model of the BBB was initially assessed for Apical to Basal transport (A to B, blood-to-brain side). Transport to the Basal side, expressed as a percentage of CoQ_10_ in the Apical compartment, was undetectable at 30 min (Figure 1a). After 2 h, the percentage in the Basal compartment rose to 0.51 ± 0.15%, but was still lower than the paracellular marker FITC-40 which was 1.03 ± 0.17% (*n* = 4). Based on data that CoQ_10_ is carried by lipoproteins in blood, CoQ_10_ was pre-incubated in serum (BPDS) for 45 min for adsorption of lipophilic CoQ_10_ to the range of endogenous lipoproteins, and the assay repeated. Total CoQ_10_ in the Apical compartment was unchanged (10.2 ± 1.1 µM and 9.0 ± 0.7 µM CoQ_10_ respectively), but transport across the PBEC monolayer increased 4-fold (Figure 1a) suggesting that CoQ_10_ may be primarily transported as part of a lipoprotein complex.

Since lipoprotein entry to the brain is tightly regulated by both uptake and efflux transporters, CoQ_10_ transport in both directions was compared (Figure 1b schematic), A to B (blood-to-brain) and B to A (brain-to-blood). Pre-incubation of CoQ_10_ in serum enhanced transport in both directions and was seen for primary PBEC monolayers (Figure 1c) and the mouse BBB cell line, bEnd.3 (Figure 1d), without a change in paracellular permeability (Figure 1e). Interestingly, transport in the A to B direction was matched by transport in the B to A direction. This means that although transport across the BBB is possible, there may be no “net” accumulation of CoQ_10_ in the brain because of opposing transport systems.

### 3.3. CoQ_10_ Distribution in Serum Lipoprotein Fractions

The distribution of CoQ_10_ in major lipoprotein fractions was assayed in cell culture serum before (untreated) and after 45 min pre-incubation with CoQ_10_. The serum’s endogenous CoQ_10_ content was 147.5 ± 0.5 nM and the majority was incorporated in the LDL fraction (77.7%, Table 3). In serum supplemented with 10 µM CoQ_10_, content increased in all fractions; HDL, LDL and vLDL. The LDL fraction still showed the greatest association, but CoQ_10_ distribution in the vLDL fraction increased greatly from <1% to 29%. More than 92% of supplemented CoQ_10_ was recovered in the vLDL/LDL fractions, confirming that lipoproteins are the main bio-carrier of CoQ_10_ and suggesting that the transport of CoQ_10_ at the BBB will be predominately mediated by lipoprotein interactions.

### 3.4. CoQ_10_ BBB Transport: SR-B1, LDLR, and RAGE Inhibitors

Based on known transport systems at the BBB for general classes of lipoproteins, relevant pharmacological inhibitors were screened for their effect on CoQ_10_ transport across bEnd.3 cells. The inhibitors were: BLT-1, which irreversibly inhibits the HDL receptor SR-B1, a receptor that also mediates vLDL uptake in hepatocytes [41]; RAP, which is widely used to inhibit the LRP-1 transporter for LDL, but also inhibits other members of the LDLR family of transporters; the RAGE inhibitor FPS-ZM1, because of the well-documented action of RAGE to oppose LRP-1 transport involving amyloid-beta and apoE; and finally, the p-glycoprotein inhibitor, verapamil was chosen to target this ABC efflux transporter, thought to mediate CoQ_10_ efflux in other cell lines.

**Figure 2:**
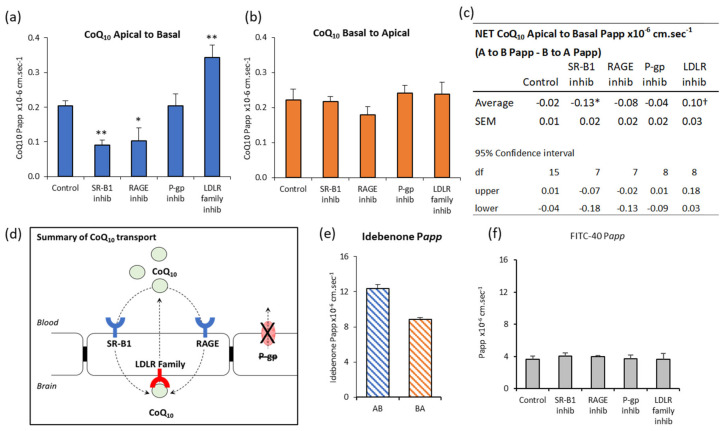
Effect of inhibitors on CoQ_10_ transport across bEnd.3 BBB model. CoQ_10_ (pre-incubated in serum) transport across bEnd.3 cells on Transwell filters assayed over 60 min. Inhibitors added apically and basally two hours before assay were BLT-1 (10 µM) for SR-B1, FPS-ZM1 (1 µM) for RAGE, receptor-associated protein (RAP) (0.5 µM) for LRP-1/LDLR and verapamil (0.1 mM) for p-glycoprotein. Apparent permeability, P*app*, shown for (**a**) Apical to Basal CoQ_10_ transport, (**b**) Basal to Apical CoQ_10_ transport. (**c**) The “net” transport of CoQ_10_ across bEnd.3 cells, calculated from the difference between A to B transport (blood-to-brain) and B to A transport (brain-to-blood). “Net” transport in control cells did not differ from zero (95% confidence interval). * Treatment with SR-B1 or RAGE inhibitors (BLT-1 10 µM, FPS-ZM1 1 µM) resulted in “net” −ve transport, i.e., “net” transport directed toward the blood side (B to A). ^†^ Treatment with LRP-1/LDLR inhibitor RAP (0.5 µM), resulted in “net” +ve transport toward the brain side (A to B). The p-glycoprotein efflux transport inhibitor Verapamil (0.1 mM) had no significant effect. (d) Schematic summary of CoQ_10_ transport across the BBB. No “net” CoQ_10_ entry toward brain side. Uptake by RAGE and SR-B1, is opposed by LRP-1/LDLR mediated removal to blood, a major impediment to brain entry of CoQ_10_. (e) Transport of the CoQ_10_ analogue, Idebenone (10 µM). Apical to basal transport exceeded basal to apical, meaning there was “net” transport toward the brain. *n* = 4-5, Values are mean ± SEM * *p* < 0.05. (f) FITC-40 Apical to Basal transport; *n* = 8 (control), *n* = 4–5 (interventions); values are mean ± SEM; * *p* < 0.05, ** *p* < 0.01; ANOVA single factor; post-hoc Bonferroni.

Inhibitors of both SR-B1 and RAGE reduced A to B CoQ_10_ transport to 44% and 50% of control respectively (Figure 2a), indicating that they normally mediate transport toward the brain. In contrast, LRP-1/LDLR inhibition with RAP revealed a 168% increase in A to B transport (Figure 2a), suggesting that this system normally opposes transport toward the brain. P-glycoprotein inhibition had no effect on transport across these cells. Interestingly, none of the inhibitors affected B to A transport (Figure 2b), which we would have expected in the case of LRP-1 inhibition by RAP. None of the interventions affected the paracellular permeability of the bEnd.3 monolayer to FITC-40 (Figure 2f), so changes in transport were not due to BBB leak or altered BBB integrity.

The “net” CoQ_10_ transport toward the brain side (A to B) is estimated from the difference between A to B transport, and B to A transport (Figure 2c). In control conditions, there is no “net” transport toward the brain side in the A to B direction (95% confidence interval, not different to zero). The only intervention to give a “net” positive transport A to B was when LRP/LDLR is inhibited with RAP, and this suggests LRP-1/LDLR is a major impediment to delivering CoQ_10_ to the brain.

By contrast, transport of the idebenone, which is an analogue of CoQ_10_, showed “net” transport toward the brain, since A to B transport exceeded B to A (Figure 2e). Compared to CoQ_10_, the permeability of idebenone (P*app*) was 280× greater in the A to B direction and 150× greater in the B to A direction. This is consistent with idebenone being able to cross the BBB directly, rather than as part of a lipoprotein, because idebenone satisfies Lipinsky’s rules for permeability as a small molecule drug (338 g/mol) with fewer than 10 hydrogen bond acceptors and LogP less than 5 [42].

Taken together, these data suggest that regulation of CoQ_10_ transport is determined by events on the blood side of the BBB, with transport systems working in opposing directions to limit the entry of lipoproteins, and therefore, limiting the entry of CoQ_10_ (Figure 2d).

### 3.5. Inhibition of CoQ_10_ Biosynthesis

**Figure 3:**
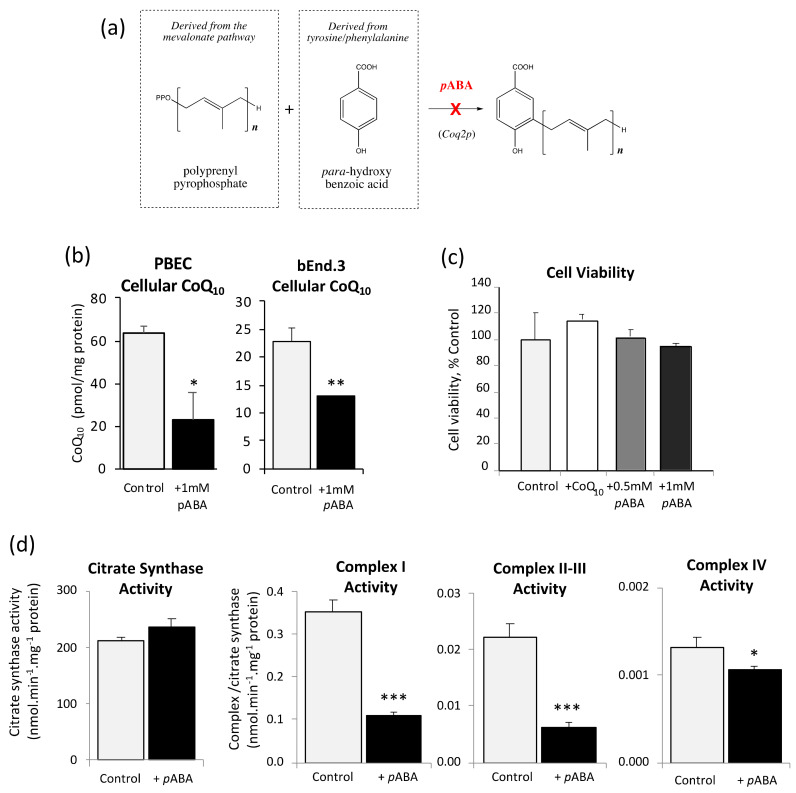
Effect of *p*ABA on cellular CoQ_10_ content, cell viability and mitochondrial respiratory chain (MRC) complexes. (**a**) Schematic showing *p*ABA inhibition of CoQ_10_ synthesis. (**b**) PBEC and bEnd.3 cellular CoQ_10_ content after treatment with 1 mM *p*ABA for 5 days (*n* = 4). (**c**) bEND.3 cell viability after 5 days treatment with CoQ_10_ (10 µM), or *p*ABA 0.5 mM or 1 mM (*n* = 6). (**d**) Effect of 1 mM *p*ABA treatment for 5 days on bEND.3 MRC complex I, II, III, and IV activity and citrate synthase (*n* = 4). Values are mean ± SEM; * *p* < 0.05, ** *p* < 0.01, *** *p* < 0.001 compared to control.

CoQ_10_ biosynthesis was inhibited by treating the BBB cells with 1 mM *p*ABA for five days before assay. As shown in Figure 3a, *p*ABA competitively inhibits polyprenyl-4-hydroxybenzoate transferase (Coq2p), a key enzyme in the latter stages of the CoQ_10_ biosynthetic pathway.

Assessment of cellular CoQ_10_ content in response to *p*ABA treatment showed significant depletion of CoQ_10_ relative to controls of 36% and 43% in PBECs and bEnd.3 cells respectively (Figure 3b). These results confirm that *p*ABA treatment induces a pronounced CoQ_10_ deficiency in the in vitro BBB, at a magnitude that is consistent with clinical presentation [36]. Despite CoQ_10_ depletion, there was no change in cell viability (Figure 3c), indicating that the cells can tolerate *p*ABA up to a concentration of 1 mM for up to 5 days, consistent with previous studies [29,43].

CoQ_10_ deficiency was also associated with a significant decrease in MRC enzyme activity across all complexes. MRC complexes I and II-III experienced the greatest relative decline in activity (68% and 72% decrease respectively, Figure 3d), with complex IV exhibiting a lesser effect (80% of control, Figure 3d). There was no significant change to citrate synthase activity (Figure 3d).

**Figure 4:**
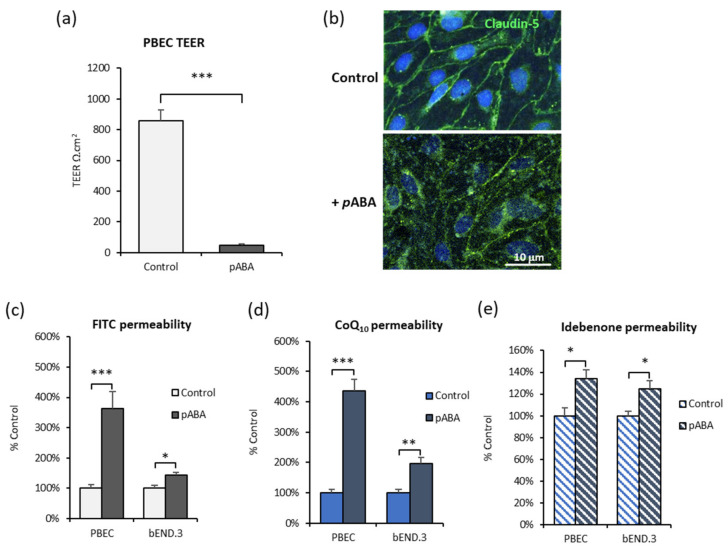
Effect of *p*ABA on in vitro BBB characteristics. BBB tight junction integrity after 5 days with *p*ABA (1 mM) treatment is compromised. (**a**) PBEC transendothelial electrical resistance (TEER) declines; *n* = 6. (**b**) Confocal microscopy staining for claudin-5 tight junction protein in PBEC cells on Transwells. Upper panel is control, lower panel after 5 days with *p*ABA (1 mM), shows reduced membrane localization of claudin-5 (40× magnification) PBEC and bEnd.3 monolayers are more leaky to (**c**) FITC-40, (**d**) CoQ_10_ and (**e**) idebenone. Values are mean ± SEM; PBEC *n* = 6, bEnd.3 *n* = 5–10; * *p* < 0.05, ** *p* < 0.01, *** *p* < 0.001.

As shown in Figure 4, BBB tight junction integrity was severely compromised after 5 days *p*ABA treatment. In PBECs, the transendothelial electrical resistance (TEER) fell from 856 ± 71 Ω · cm^2^ to 49 ± 9 Ω · cm^2^ (Figure 4a). This was accompanied by the re-location of claudin-5 tight junction protein away from the cell membrane (Figure 4b). Membrane staining was more punctate, with peri-nuclear accumulation after *p*ABA treatment, consistent with disrupted or degraded tight junctions. Both BBB cell models were leakier to the paracellular marker FITC-40 (Figure 4c), and also showed increased transport of CoQ_10_ (Figure 4d) and idebenone (Figure 4e) which is consistent with paracellular leak. A consequence of a disrupted BBB is neurological symptoms, but also, paradoxically, more CoQ_10_ could enter the brain. This would be advantageous for patients with CoQ_10_ deficiency, until sufficient CoQ_10_ was restored to the brain endothelial cells for the BBB integrity to be restored, therefore limiting further CoQ_10_ transport to the brain. This is consistent with the refractory nature of CoQ_10_ treatment, which eventually ceases being clinically effective.

### 3.6. CoQ_10_ Defecient BBB: Effect of SR-B1, LDLR, and RAGE Inhibitors

**Figure 5:**
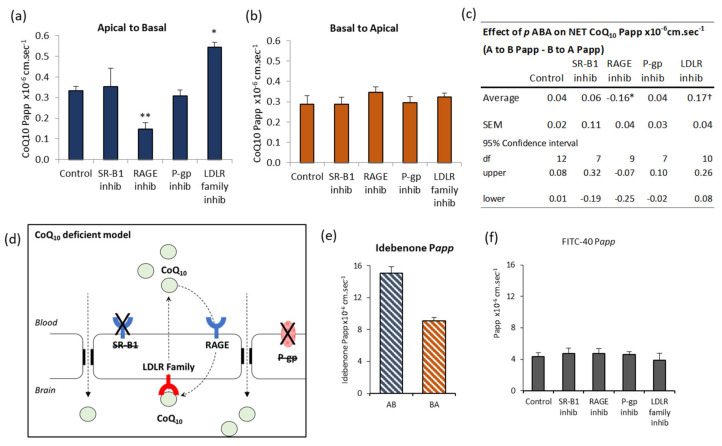
*p*ABA treated bEnd.3 cells: Effect of transport inhibitors on CoQ_10_ transport. CoQ_10_ (pre-incubated in serum) transport across *p*ABA treated (1 mM; 5 days) bEnd.3 cells on Transwell filters assayed over 60 min. Inhibitors added apically and basally two hours before assay were BLT-1 (10 µM) for SR-B1, FPS-ZM1 (1 µM) for RAGE, RAP (0.5 µM) for LRP-1/LDLR and verapamil (0.1 mM) for p-glycoprotein. Apparent permeability, P*app*, shown for (**a**) Apical to Basal CoQ_10_ transport, (**b**) Basal to Apical CoQ_10_ transport. (**c**) The “net” transport of CoQ_10_ across bEnd.3 cells, calculated from the difference between A to B transport (blood to brain) and B to A transport (brain to blood). “Net” transport in *p*ABA treated control cells was Apical to Basal, toward the brain side. * Treatment with RAGE inhibitor, FPS-ZM1 (1 µM), abolished “net” A to B transport. ^†^ Treatment with LRP-1/LDLR inhibitor RAP (0.5 µM), enhanced “net” +ve transport toward the brain side (A to B). The SR-B1 and p-glycoprotein inhibitors BLT-1 (10 µM) and Verapamil (0.1 mM), had no significant effect on “net” transport. (**d**) Schematic summary of CoQ_10_ transport across a CoQ_10_ deficient BBB. “Net” transport shifts toward to the brain side. Uptake via RAGE but SR-B1 is absent. Leaky tight junctions facilitate extra transfer across the BBB. Efflux via LRP-1 is retained. (**e**) Transport of the CoQ_10_ analogue, idebenone. Apical to Basal transport exceeded Basal to Apical, meaning there was “net” transport toward the brain. *n* = 6; values are mean ± SEM * *p* < 0.05. (**f**) FITC-40 Apical to Basal transport; *n* = 4–7; values are mean ± SEM; * *p* < 0.05, ** *p* < 0.01; ANOVA single factor; post-hoc Bonferroni.

Despite the increased paracellular leak after *p*ABA treatment, inhibitors of lipoprotein transport were able to modulate some aspects of CoQ_10_ transport, indicating that the BBB cells were still attempting to maintain homeostasis. The major difference was that SR-B1 inhibitor BLT-1, had no effect (Figure 5a), indicating that this system was inactive or ineffective after *p*ABA treatment. However, the RAGE inhibitor FPS-ZM1 reduced Apical to Basal transport to 45% of control, suggesting that RAGE was still able to transport CoQ_10_ toward the brain side. The opposing LRP-1/LDLR system also appeared active, with inhibition by RAP revealing an increased Apical to Basal transport of 164% of control (Figure 5a). As with control cells, neither Basal to Apical transport (Figure 5b) nor paracellular leak measured by FITC-40 transport (Figure 5f) were affected by the inhibitors.

Consistent with increased paracellular leak after *p*ABA treatment, the “net” CoQ_10_ transport was different to control cell “net” zero (Figure 5c), and now showed a “net” positive transport toward the brain side (“net” +ve control Apical to Basal, Figure 5c). This was dependent, in part, on the action of RAGE, since inhibiting RAGE prevented “net” transport to the brain (net −ve). Inhibiting LRP/LDLR further enhanced transport to the brain side. Transport of idebenone was also increased, consistent with increased paracellular leak, with transport toward the brain side (A to B) greater than efflux to blood (B to A) (Figure 5e).

### 3.7. Effect of Antioxidants on CoQ_10_ BBB Transport

Alongside CoQ_10_, vitamin E (α-tocopherol) is a key component of the mito-cocktail, a therapeutic mixture of potent antioxidants and cofactors administered for the treatment of mitochondrial disorders [44,45]. Both α-tocopherol and CoQ_10_ are associated with circulatory lipoprotein, and share proposed uptake mechanisms mediated by SR-B1 [46], so further experiments were designed to measure CoQ_10_ transport in the presence of α-tocopherol. As a control, this was compared to the effect of the water-soluble synthetic analogue of α-tocopherol, Trolox, which does not interact with lipoproteins but provides antioxidant activity.

**Figure 6:**
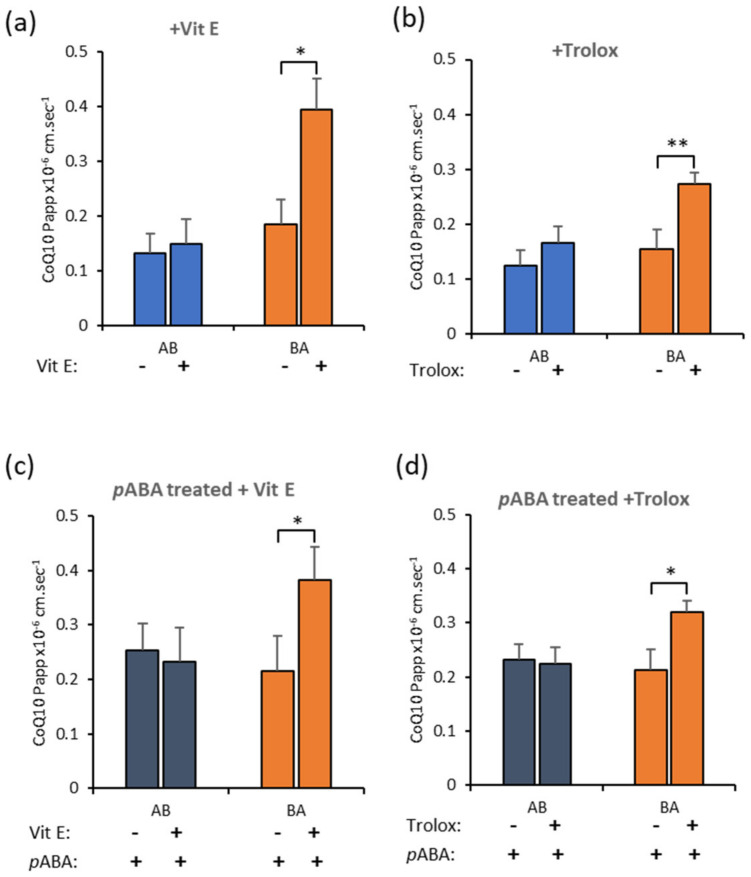
Antioxidants increase CoQ_10_ movement toward the blood side in bEnd.3 cells. Transport of CoQ_10_ across bEnd.3 monolayers on Transwell filters. CoQ_10_ (10 µM) was co-administered with either (**a**) vitamin E (50 µM) or (**b**) Trolox (50 µM) in control cells. Transport of CoQ_10_ in the direction B to A (toward blood side) was enhanced. In *p*ABA treated cells to deplete CoQ_10_, the effect persisted and both (**c**) vitamin E and (**d**) Trolox enhanced B to A transport. Values are mean ± SEM; *n* = 4–6; * *p* < 0.05, ** *p* < 0.01.

Both antioxidants—α-tocopherol (Figure 6a) and Trolox (Figure 6b)—increased Basal to Apical transport of CoQ_10_ in control conditions, i.e., toward the blood side, although they both also increased Apical to Basal transport, i.e., toward brain side, slightly. However, with *p*ABA treatment, CoQ_10_ transport toward the blood-side, dominated flux (Figure 6c,d). If this translates to clinical CoQ_10_ deficiency, then α-tocopherol co-administration with CoQ_10_ supplements would tend to reduce CoQ_10_ delivery toward the brain, the opposite of the desired effect.

## 4. Discussion

The delivery of CoQ_10_ to the brain is a crucial requirement for the clinical treatment of the CNS sequelae of CoQ_10_ deficiency. However, how CoQ_10_ might enter the brain has not been clearly defined, which makes it difficult to target treatments effectively. Animal studies indicated a certain degree of CoQ_10_ transport across the BBB. Supplementing Sprague–Dawley rat diets with 200 mg/kg CoQ_10_ for 2 months resulted in a 30% increase in cerebral cortex CoQ_10_ and CoQ_9_ (predominant ubiquinone in rat) [47]. Similarly, supplementation with high-dose (1000–5000 mg/kg) CoQ_10_ in a mouse model of Huntington’s disease significantly increased in brain levels of CoQ_10_ and CoQ_9_ [48]. However, it is uncertain from these studies whether this degree of cerebral uptake would be sufficient to replenish CoQ_10_ cellular levels in a CoQ_10_ deficient state. However, in patients with CoQ_10_ deficiency and CNS symptoms, there is limited clinical benefit of CoQ_10_ supplementation [5,8].

In this study, using an in vitro BBB model, we identify a key role for lipoproteins in CoQ_10_ transport, and illustrate that modulators of lipoprotein function determine the bi-directional transport of CoQ_10_. In the absence of serum, CoQ_10_ transport was less than that of the non-transported marker FITC-dextran, but pre-incubation of CoQ_10_ with serum before the transport assay, increased transport 4-fold in the primary porcine BBB model. Assessment of CoQ_10_ distribution in serum lipoprotein fractions was broadly similar to that of human plasma [10,49], with most CoQ_10_ in the LDL fraction, followed by HDL and vLDL. Supplementation with 10 µM CoQ_10_ increased the absolute CoQ_10_ content in all lipoprotein fractions, but proportionately more for vLDL (29%), so taken together, the increased transport across the BBB model could have been mediated by a combination of lipoproteins. Interestingly, transport in the Apical-to-Basal direction (A to B, blood-to-brain side) was matched by transport in the opposite Basal-to-Apical direction, resulting in no “net” accumulation of CoQ_10_ on the brain side because of opposing transport systems. This is consistent with the transport of lipoproteins across the BBB, which is thought to be limited because the adult brain synthesises sufficient cholesterol de novo [50], such that there is “net” efflux of cholesterol from the brain into blood.

Transport systems for lipoproteins that have been identified at the BBB include Class B scavenger receptor B1 (SR-B1), and Low-Density Lipoprotein Receptor family including LDLR and LDL receptor-related protein (LRP-1) [51,52,53]. These transporters recognise lipoproteins via their apolipoprotein (Apo) component, however, LDLR and SR-B1 recognise diverse Apo including B-100 and E for LDLR [54]; and A-I, E, and C for SR-B1 [55]. Similarly, lipoproteins may contain more than one type of Apo. For example, LDL and vLDL are rich in B-100 (LDLR ligand), and HDL is rich in Apo-A1 (SR-B1 ligand), but vLDL may also contain Apo C (SR-B1 ligand) [56], and all lipoproteins may contain Apo E (LDLR and SR-B1 ligand) [56,57]. This cross-reactivity makes attributing one type of lipoprotein to one type of transporter problematic, so for this study, we determined whether pharmacological inhibition of SR-B1 and the LDLR family of transporters could affect lipoprotein-CoQ_10_ transport overall. We also screened for two further potential CoQ_10_ transporters systems, the receptor for advanced glycation end products (RAGE) which is a brain-directed uptake transporter, and the ATP-binding cassette efflux transporter P-glycoprotein (ABCB1) which prevents entry of a range of lipid-soluble compounds to the brain.

Inhibitors of both SR-B1 and RAGE significantly reduced CoQ_10_ transport from A to B (blood to brain) by half, implicating them as important mediators of CoQ_10_ uptake to the brain. At the BBB, SR-B1 mediates transcytosis of HDL across monolayers [58] from Apical to Basal sides, and is inhibited by excess HDL (K_m_ of close to 5 µg/mL) indicative of a receptor-mediated event. In peripheral endothelial cells, SR-B1 also mediates transcytosis of LDL [59,60] in aorta ex vivo samples and coronary artery endothelial cells in culture. LDL transported across coronary endothelial cells was increased after overexpression of SR-B1 and reduced with competitive excess of LDL or HDL, which indicates a shared LDL/HDL receptor-mediated event for transport [59]. Interestingly, transcytosis in this model did not involve LDLR [59]. It was surprising that the effect of RAGE inhibition was similar to SR-B1 inhibition. RAGE is not documented to interact with lipoproteins, but is a pattern recognition receptor interacting with a broad range of negatively charged molecules [61], consistent with the anionic lipid and Apo components of lipoprotein [62], and is known to oppose LRP-1 mediated amyloid-β efflux at the BBB [17,63,64,65,66,67]. In the brain, the inhibitor FPS-ZM1 binds exclusively to RAGE with multiple down-stream effects [68], including inhibition of amyloid-β uptake, and suppression of neuroinflammation. RAGE is capable of initiating endocytosis and uptake of plasma membrane-associated molecules such as HMGB1 and S100β via caveolin [69] and lipid-raft dependent pathways [70,71]. However, studies of RAGE-mediated lipoprotein transport have not been carried out, to our knowledge.

In contrast to SR-B1 and RAGE mediated uptake of CoQ_10_, the LDLR transporters appear to oppose Apical-to-Basal uptake. Inhibition of LDLR transporters with RAP increased Apical-to-Basal uptake by 68% in the bEnd.3 BBB model. This was the only intervention to result in “net” transport toward the brain side in control cells and suggests that the LDLR family of transporters are a significant impediment to delivering CoQ_10_ to the brain. This was confirmed in our primary porcine cell model (Figure S1), where we applied RAP to the basal (brain side) of the cells, which inhibited the B-to-A efflux, so retaining CoQ_10_ on the brain side. Multiple LDLR family members may be inhibited by RAP including vLDL-R, apoE receptor 2, LDL-R, and LRP-1 [72]. LRP-1 is a possible candidate for this efflux transport because it is present on both faces of the BBB (brain and blood sides) but is responsible for the export of amyloid-β from the brain which is influenced by Apolipoproteins and inhibitable by RAP [53]. Similarly, vLDL appears to be responsible for ApoE4 associated amyloid-β efflux from the brain, although this is slower than LRP-1 mediated efflux [73]. The LDLR, in contrast, is located on the luminal (blood facing) side of the BBB and mediates transcytosis from blood toward brain in bovine brain endothelial cells [51] and in LDLR-/-mice [74]. Aside from LDLR, the other members of this family are less well studied specifically for lipoprotein transport at the BBB, and could be a fruitful avenue for future studies to improve CoQ_10_ retention by the brain. In addition, the use of LDLR inhibitors may also have potential therapeutic value as a means of increasing cellular CoQ_10_ levels in other patient groups which have been associated with a deficiency in the level of this isoprenoid, such as those with cardiovascular disease [75]. However, further studies will be required before this can be confirmed or refuted.

The final inhibitor used was for the ABC efflux transporter, P-glycoprotein (P-gp), however, no significant effect was seen on CoQ_10_ transport across the BBB. P-glycoprotein has been implicated in inhibiting CoQ_10_ uptake in the Caco-2 intestinal epithelial-barrier model [18,76], such that inhibition of P-gp improves the permeability of CoQ_10_ across the intestinal barrier. However, closer inspection of the experimental procedures indicates the use of exogenous CoQ_10_ in its pure form as opposed to being associated with lipoprotein, or in a digestive micelle, meaning the transport mechanisms described do not reflect the true in vivo environment. Furthermore, the Caco-2 intestinal barrier-model is a poor surrogate for BBB characteristics. Nevertheless, it was important to explore P-gp as a possible mode of CoQ_10_ efflux at the barrier, using a more reliable model of the BBB.

To summarise the transport assays under control conditions, the findings from this study indicate that there is a “net” efflux of exogenous CoQ_10_ from the brain to the blood in the bEnd.3 BBB model. This is the first time a receptor-mediated efflux mechanism has been implicated for CoQ_10_ at the BBB and it is in agreement with the clinical ineffectiveness of CoQ_10_ therapy for the treatment of neurological disorders [77].

The development of a CoQ_10_ deficient BBB model gives further insight into CoQ_10_ transport to the brain, and this is the first time such a model has been developed. The use of *para*-aminobenzoic acid (*p*ABA) as a pharmacological reagent to induce CoQ_10_ deficiency was first described in 1975 [28], and has since been utilised in studies of human myeloid leukemia HL-60 [43] and human neuroblastoma SH-SY5Y [29] cells. Compared with alternative techniques for inducing CoQ_10_ deficiency, for example, gene silencing [78,79,80], the use of *p*ABA, or other hydroxybenzoic acid derivates [81,82,83], is extremely cheap, very simple and highly reproducible. Building on previous findings from Duberley et al. [29], the primary porcine PBEC and murine bEnd.3 BBB cell models exhibited the depletion of CoQ_10_ to 36% and 57% of control respectively after 5 days treatment with *p*ABA (1 mmol/L). This was concomitant with a depletion of MRC enzyme activity, in particular the CoQ_10_ dependent complexes II-III (28% of control) and complex I (32% of control). However, the treatment did not correspond to a cytotoxic effect, and is consistent with previous studies [29,43]. Interestingly, and in contrast to the work of Duberley et al. [29], the activity of MRC complex IV was the least affected (80% of control), suggesting there may be a cell- or tissue-specific variation in the susceptibility of the MRC enzymes to a CoQ_10_ deficiency. Overall, however, the deficiency profile is similar to that of fibroblasts from patients with a primary CoQ_10_ deficiency, indicating that *p*ABA-treatment is an appropriate surrogate for pathophysiological investigations [9,36,84].

Under pathophysiological CoQ_10_ deficient conditions, the BBB appeared severely disrupted. Permeability of PBEC and bEnd.3 BBB models increased to both FITC-dextran and CoQ_10_, and this was accompanied by reduced tight junction integrity, measured by a drop in transendothelial electrical resistance, and the re-location of tight junction protein claudin-5 away from the cell membrane which showed punctate staining. A study by Doll and colleagues [85] showed similar effects in a bEnd.3 BBB model using mitochondrial inhibitors rotenone, FCCP and oligomycin. They found the permeability to FITC-dextran was doubled, and staining for the tight junction protein ZO-1 became punctate indicating disrupted cell–cell junctions.

Along with the increase in permeability of the pathophysiological BBB model, the “net” direction of CoQ_10_ transport was reversed compared to control. Overall, CoQ_10_ transport now favoured the blood-to-brain direction. Among the transporters studied, SR-B1 appeared non-functional, RAGE-inhibitable uptake increased from 50% to 55% of control while LDLR-inhibitable efflux reduced slightly from 68% to 63% of control, each contributing to “net” uptake of CoQ_10_ toward the brain. The overall implication is that under CoQ_10_ deficient conditions, with a disrupted BBB, transport of CoQ_10_ toward the brain is possible. This raises the possibility that restoration of normal BBB cellular CoQ_10_ levels may also restore BBB integrity, but that would also prevent further access of CoQ_10_ to the brain. This intriguing possibility may be a reason why treating CNS symptoms of CoQ_10_ deficiency is refractory in nature, becoming less effective over time.

In the final assays, we tested whether the addition of anti-oxidants could influence BBB transport of CoQ_10_, to mimic the “mito-cocktail” that is used clinically. The commonly used anti-oxidant, *RRR*-α-tocopherol had no effect on Apical to Basal uptake of CoQ_10_ (from blood to brain sides). However, there was a significant effect on efflux, which increased in both the control and pathophysiological BBB model, leading to a “net” brain-to-blood transport. This effect was surprising, and the opposite of the effect desired for improving treatment.

Since CoQ_10_ shares similar physicochemical properties to *RRR*-α-tocopherol and appears to follow analogous uptake mechanisms in vivo, including lipoprotein sequestration, we repeated the assay using a hydrophilic analog of *RRR*-α-tocopherol, Trolox, which should not interact with CoQ_10_ at the level of the lipoprotein. The result however was the same, with increased efflux, resulting in a “net” loss of CoQ_10_ from the brain side.

Of the transport systems for CoQ_10_ identified in this study, RAGE and LRP-1 function are shown to be sensitive to oxidative stress in a variety of tissue types. In general, RAGE and LRP-1 activity, or expression, increase in the presence of anti-oxidants, or in the absence of oxidative stress [86,87,88], and since these transporters are working in opposite directions across the BBB, the “net” result will be dependent upon which transporter is ultimately dominant. However, since the time-course of these experiments was relatively short (1 h), an alternative suggestion is that the reduced environment, with excess anti-oxidants, can affect lipoprotein binding to transporters, or their release after transcytosis. Indeed “reductive stress” has been implicated as a cause of BBB dysfunction and, therefore, merits further investigation given the important consequences for the treatment of CoQ_10_ deficiency [89].

## 5. Conclusions

This study demonstrated, for the first time, a dynamic interplay of multiple transport receptors, with varying degrees of influence, for the uptake and efflux of CoQ_10_ across the BBB. While there is substantial evidence for the involvement of RAGE, LRP-1 and SR-B1 in the transport of CoQ_10_ across the BBB, this is not predicted to be a comprehensive representation of all the receptors involved in its transport. The results show that the mechanisms governing uptake/efflux are complex and it is likely that there are many interactions occurring simultaneously; nevertheless, this study narrows down and isolates some key instigators, and also provides a solid foundation for further investigations.

From a clinical perspective, these findings expand our biochemical knowledge of CoQ_10_, and imply that the uptake of exogenous CoQ_10_ into the brain could be improved by the administration of an LRP-1 inhibitor, or by implementing interventions that stimulate a luminal overexpression of RAGE and SR-B1.

## Figures and Tables

**Table 1:** Comparison of performance parameters for the liquid chromatography-tandem mass spectrometry (LC-MS/MS) and HPLC-UV [20] CoQ_10_ methods.

	LC-MS/MS	HPLC-UV
LLOQ (nmol/L)	0.25	10
LLOD (nmol/L)	0.125	6
Linearity (nmol/L)	500	200
Run Time (minutes)	7	25

**Table 2:** Summary of the validation metrics for the LC-MS/MS CoQ_10_ method.

	Intra-Assay Imprecision (CV%)	Inter-Assay Imprecision (CV%)	Recovery (Ave.%)
Baseline	3.6	7.2	−
Low Spike (10 nmol/L)	5.6	6.4	84
High Spike (100 nmol/L)	5.9	−	103
EQC Plasma	−	6.7	−

**Table 3:** Distribution of CoQ_10_ in the major lipoprotein fractions.

Lipoprotein Fraction	Untreated Serum CoQ_10_ nmol/L	Supplemented Serum CoQ_10_ nmol/L
HDL	32.7 ± 1.8 (21.7%)	762 ± 8.1 (7.2%)
LDL	117.0 ± 1.2 (77.7%)	6718 ± 103.5 (63.8%)
vLDL	0.9 ± 0.01 (0.6%)	3060 ± 138.8 (29.0%)

Bovine plasma-derived serum was either untreated, or supplemented with 10 μmol/L CoQ_10_, and incubated for 45 min. Lipoprotein fractions were separated and CoQ_10_ content measured by LC-MS/MS, *n* = 3. CoQ_10_ content is given in nmol/L and the% in each fraction in parentheses. HDL, high-density lipoprotein; LDL, low-density lipoprotein; VLDL, very-low-density lipoprotein.

## Supplementary Materials

The following are available online at https://www.mdpi.com/2077-0383/9/10/3236/s1, Figure S1 (in a separate file) Key results from the bEnd.3 screen, were validated using the primary PBEC model.
