# Supplementary material for: CoQ10 Deficient Endothelial Cell Culture Model for the Investigation of CoQ10 Blood–Brain Barrier Transport
Source: J Clin Med. 2020 Oct 10;9(10):3236. doi: 10.3390/jcm9103236 (PMC7601674; doi:10.3390/jcm9103236)
Supplement: Supplementary file 1 [file jcm-09-03236-s001.pdf]

Supplementary figure

Wainwright *et al* CoQ_{10} Deficient Endothelial Cell Culture Model for the Investigation of CoQ10 Blood-Brain Barrier Transport. J Clin Med.

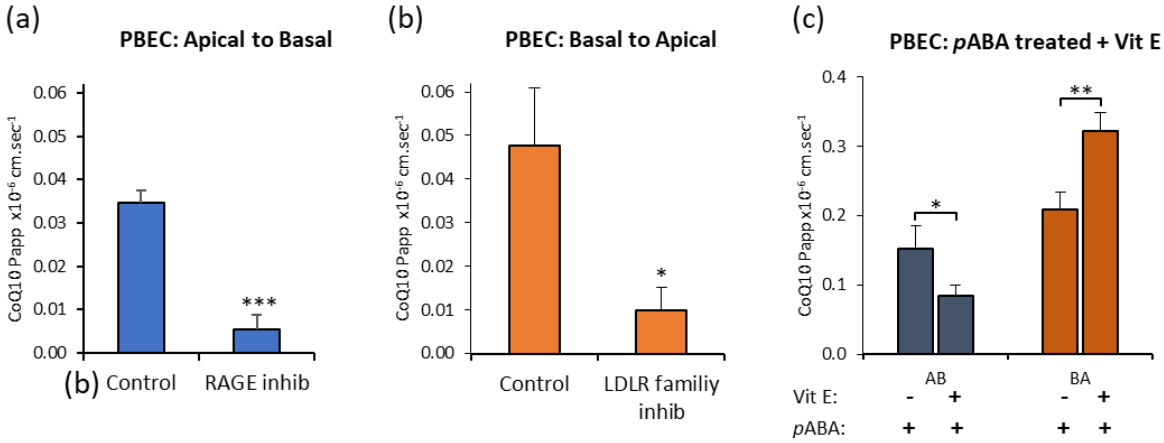

Figure S1. Key results from the b.END3 study were validated using the primary porcine brain endothelial cell (PBEC) BBB model. Effect of transport inhibitors and Vitamin E on CoQ_{10} transport **a)** from Apical to Basal using RAGE inhibitor FPS-ZM1 1µM **b)** from Basal to Apical using LDLR family inhibitor, RAP 0.5µM. **C)** Effect of Vitamin E and *p*ABA treatment on CoQ_{10} Apical to Basal and Basal to Apical transport. n=4, Values are mean ± SEM ***P<0.001;**p<0.01; *p<0.05
